# Multifaceted intervention to reduce haemodialysis catheter related bloodstream infections: REDUCCTION stepped wedge, cluster randomised trial

**DOI:** 10.1136/bmj-2021-069634

**Published:** 2022-04-12

**Authors:** Sradha Kotwal, Alan Cass, Sarah Coggan, Nicholas A Gray, Stephen Jan, Stephen McDonald, Kevan R Polkinghorne, Kris Rogers, Girish Talaulikar, Gian Luca Di Tanna, Martin Gallagher

**Affiliations:** 1 George Institute for Global Health, University of New South Wales, Sydney, NSW, Australia; 2 Prince of Wales Hospital, University of New South Wales, Sydney, NSW, Australia; 3 Menzies School of Health Research, Charles Darwin University, Darwin, NT, Australia; 4 Sunshine Coast University Hospital, Birtinya, QLD, Australia; 5 Department of Health and Behavioural Science,University of the Sunshine Coast, Sippy Downs, QLD, Australia; 6 ANZDATA Registry, South Australia Health and Medical Research Institute, Adelaide, SA, Australia; 7 Adelaide Medical School, University of Adelaide, Adelaide, SA, Australia; 8 Renal Unit, Royal Adelaide Hospital, Adelaide, SA, Australia; 9 Department of Epidemiology and Preventive Medicine, Monash University, VIC, Australia; 10 Departments of Nephrology and Medicine, Monash Medical Centre, Monash University, VIC, Australia; 11 Graduate School of Health, University of Technology Sydney, Sydney, NSW, Australia; 12 School of Population Health, University of New South Wales, Sydney, NSW, Australia; 13 Renal Services, ACT Health, Canberra, ACT, Australia; 14 South Western Sydney Clinical School, University of New South Wales, NSW, Sydney, Australia

## Abstract

**Objective:**

To identify whether multifaceted interventions, or care bundles, reduce catheter related bloodstream infections (CRBSIs) from central venous catheters used for haemodialysis.

**Design:**

Stepped wedge, cluster randomised design.

**Setting:**

37 renal services across Australia.

**Participants:**

All adults (age ≥18 years) under the care of a renal service who required insertion of a new haemodialysis catheter.

**Interventions:**

After a baseline observational phase, a service-wide, multifaceted intervention bundle that included elements of catheter care (insertion, maintenance, and removal) was implemented at one of three randomly assigned time points (12 at the first time point, 12 at the second, and 13 at the third) between 20 December 2016 and 31 March 2020.

**Main outcomes measure:**

The primary endpoint was the rate of CRBSI in the baseline phase compared with intervention phase at the renal service level using the intention-to-treat principle.

**Results:**

1.14 million haemodialysis catheter days of use were monitored across 6364 patients. Patient characteristics were similar across baseline and intervention phases. 315 CRBSIs occurred (158 in the baseline phase and 157 in the intervention phase), with a rate of 0.21 per 1000 days of catheter use in the baseline phase and 0.29 per 1000 days in the intervention phase, giving an incidence rate ratio of 1.37 (95% confidence interval 0.85 to 2.21; P=0.20). This translates to one in 10 patients who undergo dialysis for a year with a catheter experiencing an episode of CRBSI.

**Conclusions:**

Among patients who require a haemodialysis catheter, the implementation of a multifaceted intervention did not reduce the rate of CRBSI. Multifaceted interventions to prevent CRBSI might not be effective in clinical practice settings.

**Trial registration:**

Australia New Zealand Clinical Trials Registry ACTRN12616000830493.

## Introduction

Patients who require dialysis are among the groups at highest risk of healthcare associated infections.^1^ The use of central venous catheters as vascular access for haemodialysis adds to this risk through increasing the susceptibility to haemodialysis catheter related bloodstream infection (CRBSI), which is associated with higher morbidity, healthcare costs, and mortality.^2^ ^3^ Because of the many elements of haemodialysis catheter care, multifaceted interventions, or care bundles, have been postulated as an effective means to reduce this infectious burden.

Management of central venous catheters for haemodialysis (haemodialysis catheters) is complex and is perhaps best illustrated by the 20 topic areas in the US Centers for Disease Control and Prevention guidelines for the prevention of intravascular catheter related infections.^4^ In such a context, care bundles, which usually contain three to five evidence informed interventions, offer an appealing approach to reduce unnecessary clinical variation and improve patient outcomes.^5^ ^6^ The most prominent of the studies using this approach was the Michigan Keystone project, which found significant reductions (66%) in central venous CRBSIs using a before and after design in an intensive care setting.^7^ The evaluation of care bundles, however, both in general and in the specific setting of preventing central venous catheter related infections, has been limited by the paucity of evidence in a randomised setting. A 2017 meta-analysis found only six randomised studies (2049 participants) of a total of 37 studies evaluating bundles across all healthcare settings.^6^ Although the 31 before and after studies (119 178 participants) estimated a 34% risk reduction with care bundle use, the randomised trials showed no effect and the totality of evidence was graded as very low or low quality. Similarly, a 2016 meta-analysis of care bundles that focused on the outcome of preventing CRBSI found one randomised trial of 59 reports and concluded that the overall evidence quality for such interventions was low.^8^

Robust, randomised designs are especially important in evaluating care bundles. Before and after designs are particularly susceptible to the Hawthorne effect (the phenomenon whereby individuals change their practice because of their knowledge of being observed)^9^ as well as unmeasured changes in practice, whereas randomised designs can isolate these effects from the study intervention. We designed a pragmatic, national, stepped wedge, cluster randomised trial (REDUcing the burden of dialysis Catheter ComplicaTIOns—a National approach (REDUCCTION)) to examine the effect of a multifaceted, evidence based, suite of interventions on CRBSI rates in renal services across Australia (box 1 lists the components of the suite).

Box 1 **Suite of interventions**

**Recommendations for intervention implementation**

At time of catheter insertion
Surgical aseptic technique (hand hygiene, sterile gloves, surgical mask, eye protection, and gown), and a sterile environment (sterile surgical field on the patient) or a sterile room as per unit availability must be applied.
An antiseptic solution using a minimum of 2% chlorhexidine with 70% alcohol must be used
Site of insertion:
  The right internal jugular vein is the best site for catheter insertion
  Avoid catheters in the subclavian vein owing to incidence of central vein stenosis
  Avoid femoral catheters when possible
We do not recommend any specific catheter type
Ultrasound guided catheter placement is recommended if the resources are available
Semi-permeable transparent dressing must be applied to the line. If a patient is allergic to these dressings, then an alternative appropriate dressing may be used
All patients must receive education on the following topics:
  Vascular access care
  Hand hygiene
  Risks related to catheter use
  Recognising signs of infection
  Instructions for access management when away from the dialysis unit
  To ensure that their catheter and exit site are kept dry
  To seek assistance from dialysis should a dressing become wet, soiled, or leak, or if the catheter itself begins to slip out
  To not shower in the first 72 hours after catheter insertion. After 72 hours, in order to have a shower, the catheter site must be covered with waterproof material
All patients should receive a copy of the REDUCCTION catheter care sheet

**Catheter maintenance**

Hand hygiene, sterile gloves, a plastic apron, and aseptic technique (hand hygiene, gloves) must be applied at all occasions of catheter access:
  An antiseptic solution using a minimum of 2% chlorhexidine with 70% alcohol must be used
  For those unable to tolerate chlorhexidine, povidone-iodine or 70% alcohol may be used
Dressing must be changed at least every seven days and each time the dressing appears visibly soiled or loose
We do not recommend the routine use of mupirocin ointment or medicated honey at the catheter exit site
All units must use at least one of the following specific interventions aimed at prophylaxis against catheter related bacteraemia*:
  Impregnated dressings (such as chlorhexidine impregnated patch or sponge) at the catheter exit site and/or
  Antimicrobial (eg, citrate or taurolidine based) or antibacterial (eg, gentamicin) catheter locking solutions†
All patients must be advised to ensure that their catheter and exit site are kept dry. Patients must be advised to seek assistance from dialysis should a dressing become wet, soiled, or leak, or if the catheter itself begins to slip out
All patients should receive a copy of the REDUCCTION catheter care sheet
All patients must receive education on the following topics:
  Vascular access care
  Hand hygiene
  Risks related to catheter use
  Recognising signs of infection
  Instructions for access management when away from the dialysis unit
All patients must be advised not to shower in the first 72 hours after catheter insertion. After 72 hours, in order to have a shower, the catheter site must be covered with waterproof material

**Catheter removal**

Catheters must be removed as soon as it is clinically identified that they are no longer needed and within a maximum of two weeks of their last use
Non-tunnelled catheters should be changed to tunnelled catheters as soon as possible. Non-tunnelled femoral catheters should not be in place for more than five days, and non-tunnelled upper limb catheters should not be in place for more than seven days
Catheters must be removed when there are signs of catheter related infections, except in extenuating cases
Re-wiring of catheters is not recommended in the setting of any catheter related infection

*Check manufacturer’s instructions when choosing the intervention to ensure compatibility with catheters.†With the use of gentamicin locks, monitoring of antibiotic resistance should be considered as per hospital policy.

## Methods

### Study design and participants

We conducted REDUCCTION at 37 renal services across all Australian states and territories using a stepped wedge, cluster randomised design. Eligibility for renal service participation depended on available staff to collect patient data and implement the intervention. Appendix table e1 lists the participating services. The trial protocol has been reported previously, along with the statistical analysis plan.^10^ ^11^

Participating services collected data for all adults (≥18 years) receiving care from the renal service and who required a new haemodialysis catheter. The intervention was clustered and implemented at the service level; thus, all participants under the care of that unit received the same care during the intervention phase. In view of this design requirement, each site used one of two approaches to participant consent according to local research governance advice: a waiver of consent or an opt-out approach relating to data collection only. 

### Study interventions and implementation

The interventions suite covered the duration of catheter care, with elements applied at the time of insertion, during maintenance, and at removal (box 1 and appendix 4).^10^ Each service delivered the suite as a package to all patients under the care of that service, with the timing of implementation determined by randomised assignment into one of three intervention tranches. The implementation allowed services to choose if they wished to use either antibacterial dressings for a haemodialysis catheter or locking solutions, or both, but otherwise strongly encouraged the implementation of all other aspects of the intervention suite. A national guideline had been in place since 2013; the study baseline survey provides the only insight into how widely these guidelines were being incorporated into clinical practice, but it does not include data for the completeness of uptake.^12^ Baseline practice at services was measured as part of a previously reported Australian-wide and New Zealand-wide survey in the year before project initiation, and it showed wide variations in practice, especially in the catheter locking solutions and dressings used.^13^ Important elements of the project that were active from the start of the trial (baseline phase) included the requirement for clinical leaders (medical and nursing) at each service, and the entry of prospective data into a project specific, standardised, national, web based database. Measurement of the extent of implementation of the intervention was limited to recording the use of antibacterial dressings or locking solutions, or both, at three points: the time of catheter insertion, during maintenance, and at removal, in keeping with the pragmatic nature of the trial. We did a formal qualitative evaluation, which will be reported separately.

### Randomisation and masking

A covariate based constrained randomisation was used to ensure that the allocation of renal services into three intervention tranches was balanced for haemodialysis catheter use over the trial period.^11^ ^14^ ^15^ The timing and nature of the trial interventions remained confidential and was revealed six weeks before implementation at each service (appendix 4).

### Outcomes

The primary trial outcome was the comparative trial-wide service level rate of CRBSI per 1000 catheter days of use, between the baseline and intervention trial phases. Using a modified version of the Infectious Diseases Society of America definition, we defined CRBSI as any one of the following: culture of the same organism from both the catheter tip and at least one peripheral percutaneous blood culture, culture of the same organism from at least two blood samples (one from a catheter hub and the other from a peripheral vein), or bacteraemia in the absence of another (non-haemodialysis catheter) source.^16^

We had three prespecified secondary outcomes. Firstly, suspected or possible CRBSI, defined as any haemodialysis catheter removal owing to suspected infection with negative or positive blood cultures but not meeting the definition of the primary outcome, or any report from a participating service of suspected or possible CRBSI that did not meet the primary outcome definition. Secondly, total bloodstream infection rate, defined as any episode of confirmed, suspected, or possible CRBSI. Thirdly, all infectious events, including infections at exit site, the primary outcome, and individual components of secondary outcomes.

### Event adjudication and safety

Two adjudicators independently reviewed all reported infectious events. In the event of a disagreement, a third adjudicator reviewed the event and, if required, the blinded event was reviewed by the trial executive management committee and decided by consensus.

A data safety monitoring committee was not convened as the risk of harm from the intervention suite was considered low and participating services could view their rate of the trial primary outcome throughout.

### Statistical analysis

For our power calculation, we used pilot and registry data to estimate that an average of 100 haemodialysis catheter insertions occurred each year at a medium sized renal service (defined on the basis on the number of patients dialysed by the dialysis unit as reported to ANZDATA). The expected rate of baseline CRBSI was 2.5 per 1000 catheter days, derived from a combination of pilot data and published literature at the time.^17^ The intracluster correlation coefficient was estimated at 0.07 per 1000 catheter days,^18^ and the number of intervention points (number of steps) was three. Finally, on the basis of data from outcomes of the Michigan Keystone project,^7^ we assumed a 50% reduction in the risk of CRBSI from the intervention. Based on these assumptions, the trial had a power of more than 0.9 to detect a 50% reduction in the CRBSI rate with a proposed sample of 30 renal services following 100 patients for each step of the stepped wedge design.^11^

We captured all data through the REDUCCTION web based data capture system, and no provisions were made for missing data. We measured haemodialysis catheter use until the catheters were removed or no longer under the care of the participating renal service. Catheter use and events were allocated to the trial phase in which they were active. For discrete variables, we used frequencies and percentages (calculated according to the number of patients for whom data were available). For continuous variables, we used mean and standard deviation, or median and interquartile range.

We analysed our data according to the intention-to-treat principle. The primary outcome was analysed using a multilevel Poisson regression model to estimate the effect of treatment on infection rates, reported as incidence rate ratios with corresponding 95% confidence intervals for the comparative trial-wide rate at the service level of per 1000 catheter days of use between the baseline and intervention trial phases. This model included a random intercept at the service level, a fixed effect covariate for the study time periods, and an offset for log person times accumulated in the specific service and time periods.^14^ The effect of the intervention on the primary outcome was further analysed in two prespecified subgroups. Firstly, enrolling renal service size, which is a dichotomous classification of services as less than or greater than and equal to the median number of patients receiving dialysis at each service managed as of 31 December 2016. Secondly, baseline renal service practice, which is a dichotomous classification of services as concordant, or not, at trial initiation, with local guideline recommendations to use either an impregnated dressing or a locking solution as part of routine catheter care. We defined adverse events related to the intervention as the number and proportion of patients experiencing any event. All tests were two sided with a nominal level of alpha set at 5%, and we conducted the analyses using SAS software (version 9.3 or above) and R software (version 4.0.3).

### Patient and public involvement

Patients were consulted through the resources of Kidney Health Australia for their input into the intervention information sheet. Patients were not involved in the design or execution of the study, but people with a lived experience of kidney disease will be consulted to assist with the dissemination of the study results to participating sites.

## Results

### Patient, catheter, and service characteristics

From 20 December 2016 to 31 March 2020, we collected data from 6364 unique participants across 37 participating renal services (table 1; also see appendix figure e1). Overall, 3519 participants were included in the baseline phase and 2845 in the intervention phase. 

**Table 1:** Patient characteristics during baseline and intervention phases of trial

Characteristics	Baseline phase (n=3519)	Intervention phase (n=2845)
Women	1398 (39.7)	1110 (39.0)
Mean (SD) age	60.7 (15.8)	60.9 (15.9)
Ethnicity:		
Asian*	293 (8.3)	240 (8.4)
White	2250 (63.9)	1822 (64.0)
First Nations**†**	378 (10.7)	342 (12.0)
Pacific Islander**‡**	89 (2.5)	63 (2.2)
Other or not recorded	509 (14.5)	378 (13.3)
Diabetes mellitus:		
Diet controlled	304 (8.6)	172 (6.0)
Drug controlled	1251 (35.5)	1049 (36.9)
Immunosuppressant use	472 (13.4)	372 (13.1)

SD=standard deviation.

Up to 156 patients (2.3%) were transferred between centres (participating and non-participating) and have contributed more than once to characteristics. Values are numbers (percentages) unless stated otherwise.

* Includes Chinese, Malay, Filipino, Vietnamese, and Indonesian.

† Included Aboriginal and Torres Strait Islander and Māori.

‡ Included Tongan, Samoan, and Cook Islander.

The clinical characteristics of the participants were similar across the baseline and intervention phases (table 1). Across the trial population of 6364 people, participants had a mean age of 60.7 (standard deviation 15.9) years, –2508 (39.5%) were women, 2776 (43.6%) had diabetes mellitus, and 844 (13.3%) had a history of immunosuppressant treatment. A total of 11 293 haemodialysis catheters contributed to the data collection: 5431 in the baseline phase and 5862 in the intervention phase, representing 1.14 million days of haemodialysis catheter use (table 2). The major indications for insertion of a haemodialysis catheter were acute kidney injury (3763 (33.3%) catheters), or initiation of maintenance haemodialysis without permanent access (4043 (35.8%) catheters; table 2), which accounted for most catheter days of use (517 978 catheter days). A total of 8882 catheters were removed by the end of the trial, with most removed because they either were no longer required (4405 (49.6%)) or needed replacement (1550 (17.5%); appendix table e3).

**Table 2:** Characteristics of haemodialysis catheters during baseline and intervention phases of trial

Characteristics	Baseline phase			Intervention phase	
	Catheters	Catheter days		Catheters	Catheter days
Total No of catheters	5431	497 875		5862	648 390
Insertion on left side of body	1003 (18.5)	82 479		1080 (18.4)	104 582
Vein of insertion:					
Internal jugular	4653 (85.7)	457 083		5130 (87.5)	615 014
Femoral	592 (10.9)	15 058		554 (9.4)	15 034
Subclavian	152 (2.8)	21 752		139 (2.4)	13 447
Other or unknown	34 (0.7)	3982		39 (0.7)	4895
Catheter type*:					
Tunnelled	4069 (74.9)	482 001		4696 (80.1)	633 820
Non-tunnelled	1361 (25.1)	15 838		1165 (19.9)	14 297
Reason for central venous access:					
Acute kidney injury	1898 (34.9)	95 340		1865 (31.8)	118 947
Start of maintenance dialysis without functioning access	1808 (33.3)	215 685		2235 (38.1)	302 293
Transfer from peritoneal dialysis (temporary or permanent)	644 (11.8)	75 709		612 (10.4)	85 541
Arteriovenous fistula or graft thrombosis	645 (11.9)	70 056		742 (12.7)	89 175
Arteriovenous fistula or graft infection	95 (1.7)	6636		78 (1.3)	8730
Other	344 (6.1)	587		331 (5.6)	3514

Values are numbers (percentages) unless stated otherwise. *One catheter in each phase had a missing or unknown type of catheter documented, with corresponding missing catheter day counts of 36 days in baseline phase and 273 in intervention phase.

### Intervention implementation

**Fig 1:**
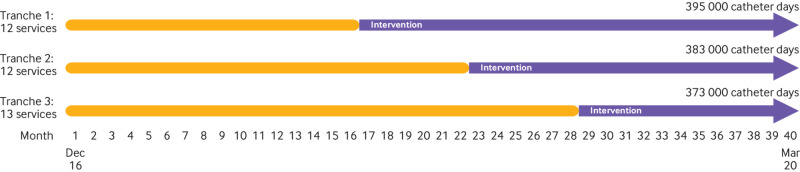
Trial timelines

The randomisation of renal services to one of the three trial intervention tranches resulted in a balance in the size of services and haemodialysis catheter use across the three tranches (fig 1; table 2; appendix table e2).

The services varied in their need and ability to change elements of their usual practice as part of the intervention. Many elements of the intervention were already being used by sites because the recommendations were included in pre-existing guidelines. For example, the introduction of an antimicrobial impregnated dressing occurred in nine (24%) of the 37 services, and three services added antimicrobial catheter locking solutions to their standard practice, whereas the remaining services already had access to antimicrobial impregnated dressings or locking solutions as part of their baseline practice. Almost all catheters (3720 (98.7%) of 3769), with a duration of more than 28 days, managed during the intervention phase involved an antimicrobial impregnated patch, sponge, or disc, and 1461 (38.8%) of 3769 involved an antimicrobial locking solution (see trial interventions section of appendix).

Other elements of the intervention were unavailable or not used previously—most notably, the ability to compare CRBSI rates using a common definition and data collection platform, the information materials for patients about catheter insertion and care, and project specific videos to train staff involved in catheter insertion and maintenance. A total of two safety events were reported in the trial, with no serious adverse events.

### Primary outcome

**Fig 2:**
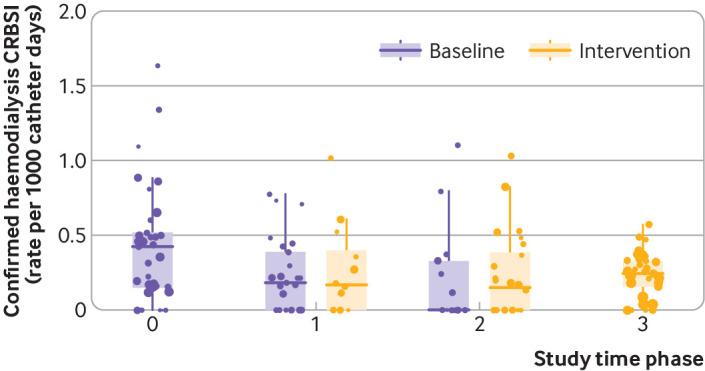
Box plot of raw rate of confirmed haemodialysis catheter related bloodstream infection (CRBSI) per 1000 catheter days, at service level, across the study. Time points are defined: 0 is all services in baseline phase; 1 is first tranche (12 services) of intervention implemented; 2 is second tranche implemented with total of 24 services in intervention phase; 3 is final tranche implemented with all services in intervention phase. Dots are weighted to size of renal services (according to local dialysis registry). Box represents interquartile range and line represents median rate during trial phase

A total of 315 episodes of confirmed CRBSI occurred during the trial: 158 in the baseline phase and 157 in the intervention phase. Table 3 shows the results of the primary outcome rates of CRBSI derived from the Poisson model and the raw event rates (also see appendix table e4), without adjustment for service level clustering and time (fig 2). The primary outcome of the service level incidence rate ratio of CRBSI from the baseline to the intervention period using the Poisson regression model was 1.37 (95% confidence interval 0.85 to 2.21; P=0.20; table 3).

**Table 3:** Trial outcomes. Rates are shown per 1000 catheter days

Outcomes	Raw event rate during baseline	Poisson mode event rate* during baseline (IQR)	Raw event rate during intervention	Poisson model event rate during intervention (IQR)	Incidence rate ratio (95% CI)	P value
Primary outcome:						
Confirmed haemodialysis CRBSI	0.32	0.21 (0.15-0.29)	0.24	0.29 (0.21-0.39)	1.37 (0.85 to 2.21)	0.20
Secondary outcomes:						
Suspected or possible CRBSI	0.13	0.12 (0.07-0.20)	0.13	0.06 (0.03-0.11)	0.52 (0.26 to 1.03)	0.06
Total CRBSI (confirmed, suspected, and possible)	0.44	0.36 (0.26-0.48)	0.37	0.35 (0.26-0.48)	0.99 (0.67 to 1.47)	0.97
All haemodialysis CVC related infectious events	0.64	0.55 (0.41-0.74)	0.50	0.40 (0.29-0.54)	0.72 (0.52 to 1.01)	0.06

CRBSI=catheter related bloodstream infection; CI=confidence interval; CVC=central venous catheter; IQR=interquartile range.

* Multilevel Poisson regression model included a random intercept at service level, a fixed effect covariate for study time periods, and an offset for log person times accumulated in specific service and time periods.

† Includes exit site and tunnel infections.

### Secondary outcomes

A total of 144 episodes of suspected or possible CRBSI, 459 total bloodstream infections, and 643 central venous catheter related infectious events occurred during the trial. Service level incidence rate ratios did not differ between the baseline and intervention phases for suspected or possible CRBSI (0.52, 95% confidence interval 0.26 to 1.03; P=0.06), total bloodstream infections (0.99, 0.67 to 1.47; P=0.97), or all infectious events (0.72, 0.52 to 1.01; P=0.06; table 3).

### Subgroup analysis

No evidence was found of between group heterogeneity in the effect of the intervention across the two prespecified subgroups analysed: enrolling renal service size and baseline renal service practice (appendix table e5).

## Discussion

In this large, national, cluster randomised trial, we tested the effect of implementing a care bundle comprised of evidence-based interventions used by treating renal services and found that the intervention did not reduce the rate of haemodialysis CRBSIs. The use of a randomised design allowed for a robust assessment of such a care bundle by adjusting for the effect of non-study related changes in practice that occur with time. Our results suggest that the effects of such care bundles on patient outcomes are either small or non-existent in contemporary clinical settings.

### Comparisons with other studies

Previous literature on the effect of care bundles has largely used before and after designs that are subject to important biases and confounding. A meta-analysis of the literature concluded that the evidence for effect is of low quality,^6^ ^8^ and the only randomised trial examining the prevention of CRBSI (in intensive care units) did not contribute to the meta-analysis because summary results could not be derived.^19^ A broader meta-analysis did, however, highlight that the randomised studies suggested no effect from care bundles.^6^ The Hawthorne effect is well recognised as a possible driver of the large effect sizes reported in the many before and after studies, but its contribution to the effect estimates in previous studies has not been discernible from the literature. The changes in the raw CRBSI rates noted during the initial observational phase of our study (fig 2), entirely independent of the care bundle intervention, give an important indication of the scale of this effect and suggest it is consistent with that reported in the uncontrolled studies.

The rate of CRBSI in our trial was much lower than the predicted values and therefore increases the risk of our results representing a false negative result. We based our trial on reported rates of between 1.1 to 5.1 per 1000 days of catheter use, and the discrepancy probably reflects improvements in underlying practices to control infections and standards of care in recent years.^20^ ^21^ ^22^ Furthermore, given the low rates measured, it is probable that further sizeable reductions might not be possible and would be difficult to detect in future clinical studies. Given the published literature, its reliance on non-randomised data, and the addition of the data from our randomised trial, the previous signals of effectiveness of care bundles need to be viewed with caution because these interventions are not without cost and risk and any benefit might be limited in clinical practice settings.

### Strengths and limitations of this study

The REDUCCTION trial has several strengths—most notably its scale in examining 1.14 million days of haemodialysis catheter use, along with a robust randomisation process that resulted in a good balance of service characteristics across the trial. These features, along with the blinded adjudication of the primary outcome, reduce the risk of two important sources of potential bias seen in other studies, namely confounding and selection bias. In addition, the national breadth of the trial means that the findings are likely to be broadly generalisable to patients requiring the use of haemodialysis catheters in other high-middle income countries.

The trial also has limitations. It was not possible to ensure that all catheters and events from every service were entered into the trial, nor was it possible to blind renal services to the intervention or to measure the detail and extent of change in practice from the intervention at the service level. A third of sites did change substantial aspects of catheter care (dressing types and locking solutions), with high rates of adherence shown during the intervention phase. Although many elements of the intervention were part of the existing clinical practice at services, some key elements were not available before the study, such as designated clinical leaders, the ability to access real time rates of CRBSI at a service and national level, and the education tools for patients and clinicians.

In addition, the event rate for the primary outcome was much lower than that of the forecast and this reduced the power of the study to detect an effect from the intervention.^9^ Estimating an overall rate for study power was challenging because of the variation in the nature and completeness of measurement of CRBSI rates across Australia, as well as the variability in the denominators used.^10^ ^13^ These event rates were lower than expected and were probably a function of several local and broader health sector initiatives aimed at reducing healthcare associated bloodstream infections over the past decade. However, the absence of detailed national data for CRBSI event rates and practice changes over time meant it was difficult to provide a definitive explanation for the lower event rates in our trial, and to exclude the risk that participating sites represent a high performing subset of national renal services.

### Implications for practice and future research

Although a CRBSI rate of 0.3 episodes per 1000 catheter days is considered low, it does equate to about one in 10 patients who undergo dialysis with a haemodialysis catheter for a year experiencing an episode of CRBSI. Such a disease burden has substantial implications for patients’ morbidity and mortality, as well as for health services with higher catheter usage, and motivates us to further study factors and treatments that might mitigate this burden. The distinction as to whether it is the overall project or the specific intervention that is affecting change in outcomes could be viewed as esoteric. Such a view, however, ignores the fact that care bundles can result in large additional costs, as seen in the challenges that many of the participating renal services had in deciding on the affordability of additional dialysis catheter dressings. Furthermore, such care bundles can add more steps to care processes that are already complex, and these steps could have unpredictable ancillary impacts, which are rarely measured, on clinical services and their patients.

A major challenge in studies assessing the impact of bundles is measuring the degree of practice change, especially using a pragmatic trial design that could be implemented and sustainable. Although our prestudy survey showed wide variation in practice, it also served to highlight important elements of dialysis catheter care and served as an impetus to change practice, independent of the study intervention. Tools to measure existing and postintervention practice, without impacting on service delivery, would be an important addition to studies of bundle implementation but are difficult to implement and might not even be possible in many clinical settings. A further research challenge in the setting of central venous catheter related infections is the low rates of CRBSI reported in our study, which, if replicated in other countries, would require much larger study sizes to detect effects and increase the cost, scale, and complexity of such studies.

### Conclusions

Our findings suggest that the pragmatic implementation of a multifaceted, evidence-based care bundle did not reduce the rate of dialysis CRBSIs in patients receiving haemodialysis with a central venous catheter in a setting of low baseline rates. By adding to a scant literature base of relevant randomised trials, these results raise broader questions about the value of such care bundles in contemporary clinical practice given that bundles can be resource intensive and increase the complexity of health systems.

**What is already known on this topic**

Haemodialysis catheters are widely used globally for vascular access in dialysis treatment and substantially increase the risk of bloodstream infections
Multifaceted interventions, often referred to as care bundles, to reduce such infections have been extensively studied
Most of the studies have been observational, however, and therefore quality of evidence has been low

**What this study adds**

Implementation of a multifaceted, evidenced based package of interventions did not alter the rate of dialysis catheter related bloodstream infection
The rate of bloodstream infection was lower than expected, but even at this lower rate one in 10 patients who have a catheter for a year will experience such an infection
This study suggests that multifaceted interventions intended to prevent a catheter related bloodstream infection might not be effective in contemporary clinical practice settings

## Data Availability

The individual patient data generated in the trial can be shared in accordance with the trial’s data sharing policy and in accordance with the local regulatory and ethical approval for the trial. The study protocol and statistical analysis plan have already been published.

## Web extra

Extra material supplied by authors

Web appendix: Study investigators, statistical methods, trial interventions, lay summary, and additional tables and figures

## Notes

Contributors: SK, SC, AC, NAG, SJ, SM, KRP, GT, and MG were responsible for the conception of the study, execution of the study, data collection, and interpretation. SK and MG drafted and revised the manuscript. KR and GLT analysed and verified the data. All authors reviewed and revised the draft of the manuscript. MG had overall final approval of the published version and SK submitted the manuscript for publication. The corresponding author (SK and MG) attests that all listed authors meet authorship criteria and that no others meeting the criteria have been omitted.

The lead author (SK) affirms that the manuscript is an honest, accurate, and transparent account of the study being reported; no important aspects of the study have been omitted; and that any discrepancies from the study as originally planned (and, if relevant, registered) have been explained.

Dissemination to participants and related patient and public communities: The study results will be disseminated to all participants through the participating sites and through our partner Kidney Health Australia (national kidney consumer body). The results statement will be reviewed by The George Institute Kidney Health Consumer panel for ease of understanding by consumers. The lay summary (still to be approved by the consumers) is included in the appendix.

Provenance and peer review: Not commissioned; externally peer reviewed.
