# Supplementary material for: Multifaceted intervention to reduce haemodialysis catheter related bloodstream infections: REDUCCTION stepped wedge, cluster randomised trial
Source: BMJ. 2022 Apr 12;377:e069634. doi: 10.1136/bmj-2021-069634 (PMC9002320; doi:10.1136/bmj-2021-069634)
Supplement: Supplementary file 1 — Web appendix: Study investigators, statistical methods, trial interventions, lay summary, and additional tables and figures [file kots069634.ww.pdf]

A multi-faceted intervention to reduce haemodialysis catheter-related blood stream infections: a cluster randomised trial – Online Appendix

1 Table of Contents

2 Study Investigators ... 2
2.1 List of Trial Investigators ... 2
2.2 Steering committee members ... 6
2.3 Trial Executive Management Committee members ... 6
2.4 Data Collection Sub-committee members ... 6
2.5 Engagement Sub-committee members ... 6
2.6 Interventions Sub-committee members ... 6
2.7 Study Adjudicators ... 6
3 Statistical methods ... 7
3.1 Randomisation methods ... 7
3.2 Details of Statistical Analysis ... 7
4 Trial interventions ... 9
4.1 Details of trial interventions ... 9
4.2 Intervention implementation ... 9
5 Lay Summary ... 10
6 Figures ... 11
6.1 Figure e1 - Trial Flow Chart ... 11
7 Appendix tables ... 12
7.1 Table e1 – Participating and Contributing entities ... 12
7.2 Table e2 – Renal Service characteristics by their phase of randomisation ... 14
7.3 Table e3 – Reasons for haemodialysis catheter removal ... 15
7.4 Table e4 – Unadjusted rates of all primary and secondary trial outcomes by trial phase ... 16
7.5 Table e5 – Sub-group analyses ... 17
8 References ... 18

2 Study Investigators

2.1 List of Trial Investigators

Institution	First name	Last name
Alice Springs Hospital	Senthil Kumar	Balakrishnan
Alice Springs Hospital	David	Fernandes
Armadale Hospital	Hemant	Kulkarni
Armadale Hospital	Casey	Light
Armadale Hospital	Jo	Ryan
Auckland Hospital	Emma	Marsh
Auckland Hospital	David	Semple
Alfred Health	Omar	Tombocon
Alfred Health	Rowan	Walker
Alfred Health	Scott	Wilson
Austin Health	Vilma	Lleva
Austin Health	Lucy	Mwangi
Austin Health	Peter	Mount
Austin Health	Maree	Ross-Smith
Austin Health	Marieke	Veenendaal
Barwon Health	Vicki	Smith
Barwon Health	Christine	Somerville
Cairns Hospital	Shaun	Davidson-West
Cairns Hospital	Natalie	Grainer
Cairns Hospital	Stella	Green
Cairns Hospital	Murty	Mantha
Cairns Hospital	Kati	Thiessen
Canberra Hospital	Girish	Talaulikar
Canberra Hospital	Alison	Winsbury
Canberra Hospital	Irene	Yao
Canberra Hospital	Emily	Neville
CARI	Pamela	Lopez-Vargas
Concord Hospital	Khalilah	Marquez
Concord Hospital	Mona	Razavian
Concord Hospital	Lisa	Tienstra
Concord Hospital	Glenn	Stewart
Eastern Health	Cathy	Chan
Eastern Health	Peta	McLean
Eastern Health	Lawrence	McMahon
Eastern Health	Matthew	Roberts
Eastern Health	Dong	Wang
Fiona Stanley Hospital	Monika	Chang
Fiona Stanley Hospital	Anna	Chiam
Fiona Stanley Hospital	Duncan	Wright
Fiona Stanley Hospital	Orla	O'Brien
Fiona Stanley Hospital	Ramyasuda	Swaminathan

Institution	First name	Last name
Fiona Stanley Hospital	Samadhi	Wimalasena
Fiona Stanley Hospital	Harish	Puttagunta
Flinders Medical Centre	Jeffrey	Barbara
Flinders Medical Centre	Amanda	Luke
Flinders Medical Centre	Margaret	Pummeroy
Flinders Medical Centre	Kim	Torpey
Gold Coast Hospital and Health Service	Gemma	Nicholls
Gold Coast Hospital and Health Service	Amy	Swinbank
Gold Coast Hospital and Health Service	Thomas	Titus
John Hunter Hospital	Peter	Choi
John Hunter Hospital	Ginger	Chu
John Hunter Hospital	Leanne	Garvey
John Hunter Hospital	Alastair	Gillies
Kidney Health Australia	Shilpa	Jesudason
Liverpool Hospital	Josephine	Chow
Liverpool Hospital	Imelda	De Guzman
Liverpool Hospital	Jeanny	Gando
Liverpool Hospital	Jeffrey	Wong
Liverpool Hospital	Richard	Nguyen
Mackay Hospital	Roy	Cherian
Mackay Hospital	Raye	Gillard
Mackay Hospital	Rachel	James
Mater Hospital	Michael	Burke
Mater Hospital	Leanne	Glancy
Mater Hospital	Shimbie	Lewis
Mater Hospital	Richard	Baer
Mater Hospital	Sophie	Wade
Monash Health	Kate	Fitt
Monash Health	Peter	Kerr
Monash Health	Kevan	Polkinghorne
Monash Health	Mechelle	Seneviratne
Nepean Hospital	Muralikrishna	Komala
Nepean Hospital	Junie	McCourt
Nepean Hospital	Craig	Lawlor
Ipswich Hospital	Julia	Bell
Ipswich Hospital	David	Johnson
Prince of Wales Hospital	Michaela	Kelleher
Prince of Wales Hospital	Sradha	Kotwal
Princess Alexandra Hospital	Amanda	Coburn
Princess Alexandra Hospital	Sarah	Guo
Princess Alexandra Hospital	David	Johnson
Princess Alexandra Hospital	Joanna	Sudak
Princess Alexandra Hospital	Diana	Leary
Rockhampton Hospital	Jenny	Anderson

Institution	First name	Last name
Rockhampton Hospital	Thin	Han
Rockhampton Hospital	Tresna	Titmarsh
Royal Adelaide Hospital	Emily	Adam
Royal Adelaide Hospital	Bronwyn	Hockley
Royal Adelaide Hospital	Jenny	Latte
Royal Adelaide Hospital	Yvonne	Matthew
Royal Adelaide Hospital	Stephen	McDonald
Royal Adelaide Hospital	Chen Au	Peh
Royal Adelaide Hospital	Rebecca	Taylor
Royal Brisbane Hospital	David	McIntyre
Royal Brisbane Hospital	Sharadchandra	Ratanjee
Royal Darwin Hospital	Karolynn	Maurice
Royal Darwin Hospital	Fiona	Rettie
Royal Darwin Hospital	Madhivanan	Sundaram
Royal Darwin Hospital	Naomi	Grimshaw
Royal Hobart Hospital	Matthew	Jose
Royal Hobart Hospital	Gail	Read
Royal Melbourne Hospital	Jayne	Amy
Royal Melbourne Hospital	Patricia	Coutts
Royal Melbourne Hospital	Maria	Presno
Royal Melbourne Hospital	Nigel	Toussaint
Royal North Shore Hospital	Debbie	Knagge
Royal North Shore Hospital	Colleen	Van Senden
Royal North Shore Hospital	Linh	Pham
Royal North Shore Hospital	Muh Geot	Wong
Royal Prince Alfred Hospital	Jane	Nicholson
Royal Prince Alfred Hospital	Paul	Snelling
Sir Charles Gairdner Hospital	Neil	Boudville
Sir Charles Gairdner Hospital	Alison	Farmer
Sir Charles Gairdner Hospital	Ingrid	Holmes
Sir Charles Gairdner Hospital	Victoria	Link
Sir Charles Gairdner Hospital	Vivien	Perreau
Sir Charles Gairdner Hospital	Nicole	Warnecke
St George Hospital	Sunil	Badve
St George Hospital	Yanella	Martinez-Smith
St George Hospital	Jayson	Catiwa
St Vincent's Hospital	Frank	Ierino
St Vincent's Hospital	Emmet	O'flaherty
Sunshine Coast Hospital and Health Service	Nicholas	Gray
Sunshine Coast Hospital and Health Service	Gerald	Hilder
Sunshine Coast Hospital and Health Service	Kaylene	Wadd
Sunshine Coast Hospital and Health Service	Andrea	Pollock
Sunshine Coast Hospital and Health Service	Stanley	Searle
Tamworth Hospital	Cheryl	Wertheim

Institution	First name	Last name
Tamworth Hospital	Stephen	May
Tamworth Hospital	Jill	Telfer
The George Institute for Global Health	Martin	Gallagher
The George Institute for Global Health	Sradha	Kotwal
The George Institute for Global Health	Sarah	Coggan
The George Institute for Global Health	Casey	Yates
The George Institute for Global Health	Kathryn	Higgins
The George Institute for Global Health	Earl	James
The George Institute for Global Health	Alison	Coenen
The George Institute for Global Health	Kris	Rogers
The George Institute for Global Health	Gian Luca	Di Tanna
The George Institute for Global Health	Jayanthi	Mysore
The George Institute for Global Health	Joseph Alvin	Santos
The George Institute for Global Health	Benjamin	Talbot
The George Institute for Global Health	Katrina	Thistlethwaite
The George Institute for Global Health	Bruce	Neal
Toowoomba Hospital	Elizabeth	Coroneos
Toowoomba Hospital	Shannon	Nugent
Toowoomba Hospital	Ian	Fox
Toowoomba Hospital	Sree	Venuthurupalli
Western Health	Jennifer	Connor
Western Health	Ruth	Thachaw
Western Health	Sandra	Crikis
Wollongong Hospital	Pauline	Byrne
Wollongong Hospital	Karumathil	Murali
Wollongong Hospital	Hicham	Cheikh Hassan
Western Sydney Local Health District	Lin	Huang
Western Sydney Local Health District	Romiereeza	Dizon
Western Sydney Local Health District	Deepika	Joshi
Western Sydney Local Health District	Jon	Mendoza
Western Sydney Local Health District	Bishi	Augustine
Western Sydney Local Health District	Disha	Balraj
Western Sydney Local Health District	Vincent	Lee
Western Sydney Local Health District	David	O'Donnell

2.2 Steering committee members

All above listed site investigators participated in the Steering Committee.

2.3 Trial Executive Management Committee members

Martin Gallagher; Sradha Kotwal; Sarah Coggan; Kevan Polkinghorne; Nicholas Gray; Girish Talaulikar; Alan Cass; Stephen McDonald; Stephen Jan

2.4 Data Collection Sub-committee members

Stephen McDonald; Sradha Kotwal; Sharad Ratanjee; Debbie Knagge, Marieke Veenendaal, Paul Snelling

2.5 Engagement Sub-committee members

Nicholas Gray; Sradha Kotwal; Girish Talaulikar; Suda Swaminathan; Leanne Brown; Karolynn Maurice; Maree Ross-Smith; Steve May

2.6 Interventions Sub-committee members

Kevan Polkinghorne; Sradha Kotwal; David Johnson; Emma Marsh; Madhivanan Sundaram; Peter Mount; Vincent Lee; Suda Swaminathan; Pamela Lopez-Vargas (representative of Caring for Australasians with Renal Impairment).

2.7 Study Adjudicators

Ben Talbot; David Semple; Peter Kerr; Matthew Roberts

The data collection tool was custom made for the REDUCCTION study and was developed by The Project Factory (https://www.theprojectfactory.com/) together with The George Institute study team.

3 Statistical methods

3.1 Randomisation methods

The participating services were divided into three balanced tranches using a covariate-based constrained randomisation over the trial intervention period. The allocation sequence was informed by the total number of catheters inserted in the baseline phase of the trial (December 2016 - January 2018). One hundred thousand random allocation sequences were generated, and those that achieved a degree of balance of no more than a 10% difference from the average number of dialysis catheters in the trial (between 73.2 - 89.8 average catheters per arm) were retained. Of the original sequences generated, 6,865 met our balance criteria, and the final randomisation allocation was chosen randomly from this subset.

3.2 Details of Statistical Analysis

From the published Statistical Analysis Plan.[1]

The primary endpoint is the rate of infection events per 1,000 catheter days recorded by services. A generalised linear mixed model (a modified version of model-based approach)[2] was used to estimate the effect of treatment on infection rates:

$$\log{(y_{ij})} = \mu + a_i + \beta_j + X_{ij}\theta + \log(PT_{ij})$$

i.e. a multilevel Poisson regression.

Where Y_{ij} is the number of infections in cluster *i* at time *j* ($y_{ij} = \sum_{k \in ij} y_{ijk}$).

α_i a service level random intercept. This is the difference of the infection rate of each service from the overall rate μ.

PT_{ij} is the accumulated person-time in service *i* and time *j*. This was calculated from individual patient data ($PT_{ij} = \sum_{k \in ij} PT_{ijk}$). Patient time is the number of days between the latest data of either a) beginning of the time-period or b) entry of a patient to the service; and the earliest of a) end of time-period, b) death of the patient, c) exit of the patient from the service.

β_j is fixed effect for time (classification variable with baseline time period as reference).

X_{ij} is an indicator variable for service *i* receiving the intervention at time *j*, with θ the effect estimate of receiving the intervention vs. receiving the control.

The two distributions are:

$y \sim \text{Poisson}(\lambda)$

$\alpha \sim \text{Normal}(0, \sigma^2)$

Distribution was selected based on AIC and BIC, with Poisson having the best fit according to both criteria over the negative binomial distribution. The distribution of residuals was checked with hanging rootogram plots and showed a good fit.

4 Trial interventions

4.1 Details of trial interventions

The interventions, developed by a trial Sub-committee, were informed by the most current evidence at the time of design, including the relevant Australian guideline, and were finalised in January 2018. A physician and nurse were identified at each renal service who were the local champions and were tasked with implementing the intervention at their service. A Data Safety Monitoring Committee was not convened for the project as the risk of harm from the interventions was considered low and participating services could view their rate of the trial primary outcome throughout. Local infection control processes were used to monitor any anti-microbial resistance.

4.2 Intervention implementation

The use of antimicrobial dressings and locking solutions are reported in catheters that remained in situ for 28 or more days during the intervention phase. 3720 out of 3769 such catheters had an antimicrobial impregnated patch, sponge or disc applied while 1461 of the 3769 catheters used an antimicrobial locking solution.

5 Lay Summary

People with kidney failure can be treated with haemodialysis. Blood is filtered through a dialysis machine to remove toxins and water from the blood. Filtered blood is then returned to the body. Dialysis needs access to the bloodstream. Catheters are often used for this access.

A catheter is a soft plastic tube inserted under the skin into a vein in the neck or chest. The biggest concern with catheter use is the serious risk of catheter-related blood stream infection.

The REDUCCTION study aimed to reduce the number of infections in patients who have catheters for dialysis. The trial introduced a standard process of care across the 37 kidney units in Australia who took part in this research. The study collected information on catheter use and infections for 12-18 months. Then the team introduced a standard process of care at all the sites over 18 months in a randomised fashion. The same information was collected from this point on for a further 12-18 months. The rate of catheter-related blood stream infections before and after the standard process of care were compared.

The study showed that the standard process of care did not reduce the number of infections. The rate of these infections is low in Australia. Despite this low rate, 1 in 10 patients who have a catheter for a year, will develop an infection.

6 Figures

6.1 Figure e1 - Trial Flow Chart

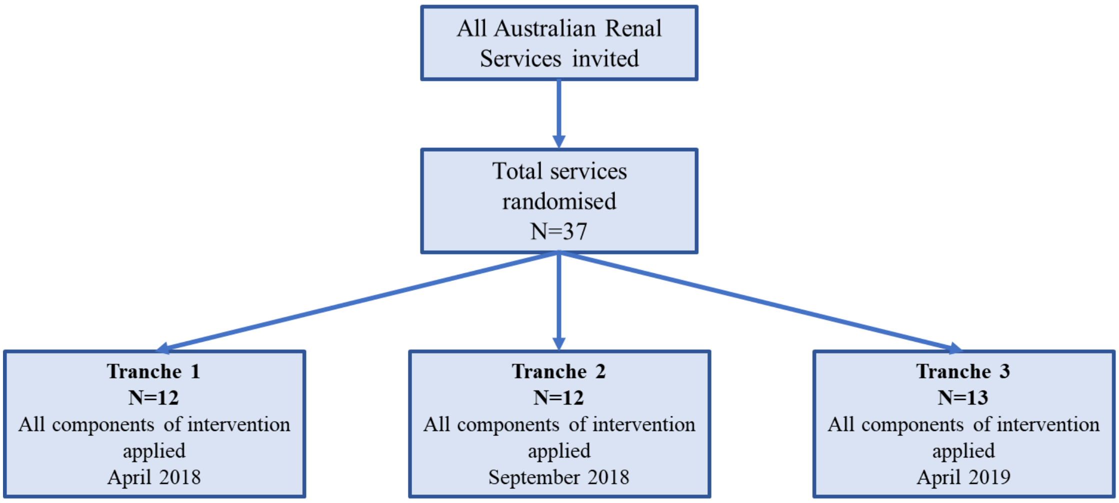

7 Appendix tables

7.1 Table e1 – Participating and Contributing entities

**Partnering organisations**
ANZDATA Registry
Department of Health and Human Services Victoria – Safer Care Victoria
Kidney Health Australia
Kidney Health Australia- Caring for Australasians with Renal Impairment
Queensland Health
**Participating Renal Services**
Alice Springs Hospital, Northern Territory
Alfred Health, Victoria
Armadale Hospital, Western Australia
Austin Health, Victoria
Barwon Health, Victoria
Cairns Hospital, Queensland
Canberra Hospital, Australian Capital Territory
Concord Hospital, New South Wales
Eastern Health, Victoria
Fiona Stanley Hospital, Western Australia
Flinders Medical Centre, South Australia
Gold Coast Hospital and Health Service, Queensland
John Hunter Hospital, New South Wales
Liverpool Hospital, New South Wales
Mater Hospital, Queensland
Mackay Hospital, Queensland
Metro South Hospital and Health Services, Queensland
Monash Health, Victoria
Nepean Hospital, New South Wales
Prince of Wales Hospital, New South Wales
Rockhampton, Queensland
Royal Adelaide Hospital, South Australia
Royal Brisbane and Women’s Hospital, Queensland
Royal Darwin Hospital, Northern Territory
Royal Hobart Hospital, Tasmania
Royal Melbourne Hospital, Victoria
Royal North Shore Hospital, New South Wales
Royal Prince Alfred Hospital, New South Wales
Sir Charles Gairdner Hospital, Western Australia
St George Hospital, New South Wales
St Vincent’s Hospital, Victoria
Sunshine Coast Hospital and Health Service, Queensland
Tamworth Hospital, New South Wales
Toowoomba Hospital, Queensland

Western Health, Victoria
Western Sydney Local Health District, New South Wales
Wollongong Hospital, New South Wales

7.2 Table e2 – Renal Service characteristics by their phase of randomisation

	Tranche 1 (n=12)	Tranche 2 (n=12)	Tranche 3 (n=13)	Overall (n=37)
**Location**				
Metropolitan	11 (91.7%)	8 (66.7%)	10 (76.9%)	29 (78.4%)
Regional	1 (8.3%)	4 (33.3%)	3 (23.1%)	8 (21.6%)
**No of Satellite sites**				
More than 3	5 (41.7%)	6 (50.0%)	5 (38.5%)	16 (43.2%)
Less than or equal to 3	7 (58.3%)	6 (50.0%)	8 (61.5%)	21 (56.8%)
**Renal Service size**				
Total dialysis patients as of 31 Dec 2016	2,956	2,646	2,896	8,498
N of catheters inserted per 12 months in baseline period*	90.2 (50.7; 127.9)	90.9 (62.8; 106.7)	78.9 (57.5; 128.2)	84.8 (57.5; 114.6)
N of catheters inserted per 12 months in intervention period*	59.6 (39.5; 145.4)	75.8 (41.9; 105.0)	68.7 (51.1; 113.5)	71.2 (42.7; 116.3)
Total catheter days in each tranche across whole trial	397,201	378,601	375,2463	1,146,265
**Changes made to standard practice**				
Number of services that changed dressings n (%)	6 (50.0%)	1 (8.3%)	2 (15.4%)	9 (24.3%)
Number of services that changed locking solutions n (%)	0 (0.0%)	2 (16.7%)	1 (7.7%)	3 (8.1%)

*reported as medians with interquartile ranges.

7.3 Table e3 – Reasons for haemodialysis catheter removal

	Baseline phase	Intervention phase
**Reason for Haemodialysis catheter removal***	**N=4,302**	**N=4,580**
No longer required	2089 (48.6%)	2316 (50.6%)
Poor catheter flow	450 (10.5%)	435 (9.5%)
Extruded catheter cuff	61 (1.4%)	74 (1.6%)
Suspected or confirmed Exit site infection	24 (0.6%)	30 (0.7%)
Confirmed sepsis	150 (3.5%)	159 (3.5%)
Possible sepsis	152 (3.5%)	145 (3.2%)
Changed catheter site/type to reduce complication risk	813 (18.9%)	739 (16.1%)
Patient discharge from renal service	179 (4.2%)	192 (4.2%)
Patient death with catheter in-situ	309 (7.2%)	350 (7.6%)
Other	75 (1.7%)	140 (3.1%)

*A total for 8,882 catheters were removed during the course of the trial. The reasons for catheter removal reported here were as reported by the renal services at the time of catheter removal and prior to the adjudication process. Although the recommendations for management of haemodialysis catheter related blood stream infection were for the haemodialysis catheter to be removed, the final decision rested with the clinical team caring for the patient.

7.4 Table e4 – Unadjusted rates of all primary and secondary trial outcomes by trial phase

	Time point	Events / Time at risk (catheter days) in baseline group	Event rate during baseline (/1,000 catheter days)	Events / Time at risk (catheter days) in intervention group	Event rate during intervention (/1,000 catheter days)
Confirmed HD-CRBSI	0	122/314,742	0.39		
	1	22/117,311	0.19	18/64,522	0.28
	2	14/65,822	0.21	38/134,279	0.28
	3			101/449,589	0.23
Suspected/possible CRBSI	0	36/314,742	0.11		
	1	19/117,311	0.16	2/64,522	0.03
	2	7/65,822	0.11	19/134,279	0.14
	3			61/449,589	0.14
Confirmed and suspected/possible CRBSI	0	158/314,742	0.50		
	1	41/117,311	0.35	20/64,522	0.31
	2	21/65,822	0.32	57/134,279	0.42
	3			162/449,589	0.36
All infectious events	0	218/314,742	0.69		
	1	62/117,311	0.53	22/64,522	0.34
	2	40/65,822	0.61	71/134,279	0.53
	3			230/449,589	0.51

Time points are defined as 0 – all services in baseline phase; 1 – first tranche (12 services) of intervention implemented; 2 – second tranche implemented with total of 24 services in intervention phase; 3 – final tranche implemented with all services in intervention phase; HD-CRBSI: Haemodialysis catheter-related blood stream infection; CRBSI: catheter-related blood stream infection

7.5 Table e5 – Sub-group analyses

	Confirmed HD-CRBSI events in baseline phase/1000 catheter days[1]	Confirmed HD-CRBSI events in intervention phase/1000 catheter days	Rate Ratio (95% CI)	*P* value for interaction
**Enrolling renal service size**				
Greater than or equal to the median (<63)	0.34	0.27	1.46 (0.88 - 2.43)	0.34
Below the median (>63)	0.27	0.19	1.14 (0.62 - 2.08)	
**Baseline renal service practice**				
Concordant with using a dressing or locking solution	0.27	0.22	1.38 (0.83 - 2.30)	0.61
Not concordant with using a dressing or locking solution	0.46	0.30	1.22 (0.68 - 2.17)	

[1] Total number of events (one catheter can contribute more than one event); HD-CRBSI: Haemodialysis catheter-related blood stream infection; CI: confidence interval

8 References

1. Kotwal S, Gallagher M, Rogers K, Di Tanna GL. REDUcing the burden of dialysis Catheter ComplicaTIOns: a National approach (REDUCCTION) - statistical analysis plan. https://osf.io/gnw3u/. Published 2020. Accessed Dec 15, 2020.
2. Hussey MA, Hughes JP. Design and analysis of stepped wedge cluster randomized trials. *Contemporary clinical trials.* 2007;28(2):182-191.
